# Microwave Assisted Rapid and Green Synthesis of Silver Nanoparticles Using a Pigment Produced by *Streptomyces coelicolor* klmp33

**DOI:** 10.1155/2013/341798

**Published:** 2013-08-31

**Authors:** Deene Manikprabhu, K. Lingappa

**Affiliations:** Department of Microbiology, Gulbarga University, Gulbarga, Karnataka 585106, India

## Abstract

Traditional synthesis of silver nanoparticles using chemical methods produces toxic substances. In contrast biological synthesis is regarded as a safe and nontoxic process but the major drawback of biological synthesis is, this process is slow. In the present investigation, we developed a rapid and green synthesis of silver nanoparticles employing a pigment produced by *Streptomyces coelicolor* klmp33 in just 90 s. The silver nanoparticles were characterized by UV-visible spectroscopy, transmission electron microscopy (TEM), X-ray diffraction (XRD), and Fourier transform infrared spectroscopy (FTIR). The biobased synthesis developed in this method is a safe, rapid, and appropriate way for bulky synthesis of silver nanoparticles.

## 1. Introduction

Nanotechnology is expected to be the basis of many important technological innovations in the 21st century [1]. Various physical and chemical methods were reported for the synthesis of silver nanoparticles, but most of these methods cause potential environmental and biological hazards [2]. Compared to physical and chemical methods, biological synthesis using microbes and plants was regarded as a safe and ecofriendly process [3]. Several biological synthesis methods, using microbes like *Cladosporium cladosporioides* [4] and *Fusarium oxysporum* [5], have been suggested as safe, cost-effective, possible ecofriendly ways and alternatives to chemical and physical methods, but these methods also have the drawback that these processes were rather slow [6]. Parallel to microbes mediated synthesis, several rapid plant mediated synthesis method using crude plant parts extracts like *Sorbus aucuparia* [7] and *Chenopodium album* [8] were also reported, but large scale usage of plants for industrial purpose synthesis may lead to loss of valuable species [9]. Therefore, there is a need to develop a rapid and ecofriendly process for the synthesis of silver nanoparticles.

In our earlier study, we reported synthesis of silver nanoparticles using pigment produced by *Streptomyces coelicolor* klmp33 by photoirradiation method in 20 min, but still we think the synthesis is more time consuming [10]. So, we explored different methods using the same pigment produced by *S. coelicolor* klmp33 to overcome this problem. Among different methods, microwave assisted synthesis showed promising result; the advantage of microwave irradiation over conventional biological synthesis is the improvement in rate kinetics of the reaction due to rapid heating and penetration involved, which may result in a narrow distribution of the particle size [11, 12].

Synthesis of silver nanoparticles using this method shows surprising results; the silver nanoparticles were synthesized within 90 s.

## 2. Experimental

### 2.1. Isolation and Identification of *S. coelicolor* klmp33

The *S. coelicolor* klmp33 was isolated from the soil sample of Gulbarga, India, and identified based on 16S rRNA sequencing, and the sequences obtained were submitted to Gene Bank under the accession number JQ27722. The *S. coelicolor* klmp33 grows best at 27°C and produces a blue pigment after 3 days of incubation.

### 2.2. Analysis and Solubility of the Pigment

*S. coelicolor* klmp33 culture was grown in starch casein medium. The blue pigment produced after 3 days of incubation was separated from the biomass by centrifugation at 10,000 rpm for 10 minutes. The resultant pigment was assayed using thin layer chromatography conducted on silica-gel 60 F_254_ plates (Merck) with benzene acetic acid (9 : 1; v/v) as solvent. The pigment was obtained as a single spot with an Rf value of 0.28 indicating the presence of gamma actinorhodin (analogue of actinorhodin).

The pigment was soluble in chloroform and methanol but insoluble in dioxane. The solubility result correlates with gamma actinorhodin [13].

### 2.3. Synthesis of Silver Nanoparticles

For the synthesis of silver nanoparticles, different volumes of different concentrations of silver nitrate solution (10^−3^ M and 10^−2^ M) were treated with 1 mL of the pigment (1 : 1, 2 : 1, 3 : 1, 4 : 1, 5 : 1, 6 : 1, 7 : 1, 8 : 1, 9 : 1, 10 : 1, 11 : 1, 12 : 1, 13 : 1, 14 : 1, 15 : 1, 16 : 1, 17 : 1, 18 : 1, 19 : 1, and 20 : 1) and exposed to microwave irradiation at a fixed frequency of 2.45 GHz. Based on the results obtained we followed the below mentioned protocol: 

20 mL of 10^−3^ M silver nitrate was treated with 1 mL of pigment and exposed to microwave irradiation for different time periods.

### 2.4. Characterization of Silver Nanoparticles

 The characterization of the silver nanoparticles was preliminary carried out using UV-visible spectroscopy (Systronics 2200 double beam UV-visible spectrophotometer). The UV-visible analysis was carried out for a period of 90 s. The synthesis was further confirmed using X-ray diffraction (XRD) recorded using a Rigaku Ultima 4 XRD instrument. The radiation used was Cu-K*α* (0.154 nm) at 40 kV and 35 nm with scanning rate of 2°/min. 

To determine size and shape of silver nanoparticles, transmission electron microscopy (TEM) was carried out. The TEM images were obtained using a Philips CM200 instrument. Samples for this analysis were prepared by coating carbon-coated copper grids with aqueous silver nanoparticles. After 5 min, the extra solution was removed using blotting paper, and then the films on the grids were dried under IR light. 

For FTIR studies, powder of pigment and silver nanoparticles was prepared by centrifuging the pigment and silver nanoparticles solution at 10,000 rpm for 20 min. The solid residue of pigment obtained was dried completely, whereas the solid residue of the silver nanoparticles was further washed with distilled water to remove any unattached biological moieties from the surface of the nanoparticles, and the resultant residue was then dried completely. The powder samples were used for FTIR measurements, which were performed on a NICOLET iS5 with Diamond ATR. The FTIR peaks were identified and expressed in wave numbers (cm^−1^).

## 3. Results and Discussion

The traditional and most widely used methods for synthesis of metallic nanoparticles use wet-chemical procedures, which involve growing nanoparticles in a liquid medium containing various reactants, in particular reducing agents. The advantage of this method is the low cost for high volume synthesis, but the major drawbacks of this method include contamination from precursor chemicals and use of toxic solvents, which shows adverse effects on environment and human health [14]. In contrast biological synthesis using microbes and plants was regarded as a safe and ecofriendly process; recently Balaji et al. reported microbes assisted synthesis of silver nanoparticles in 78 h by using 1-week-old fungus biomass of *Cladosporium cladosporioides *[4]. Similarly Durán et al. synthesized silver nanoparticles using 6-day-old biomass of *Fusarium oxysporum *strains in 72 h [5]. Though these methods are safe and eco-friendly, these processes were rather slow and not industrially feasible, as they require high aseptic conditions [15]. To overcome these drawbacks, some rapid synthesis methods using plants and mushrooms extract were also reported. Dubey et al. used *Sorbus aucuparia* plant extract and synthesised silver nanoparticles in 15 min and showed sorbic acid and its salts as a possible reducing agent present in the extract [7]. Similarly Bhat et al. also reported rapid synthesis using mushroom extract and showed flavin as a probable reducing agent, but the major problems of these methods were separation of reducing agent from the extract [16] and large usage of plants and mushrooms for industrial purpose which may lead to loss of valuable species. In the present work we described a simple, rapid, and ecofriendly process for silver nanoparticles using blue pigment produced by *S. coelicolor *klmp33 by microwave irradiation.

**Figure 1:**
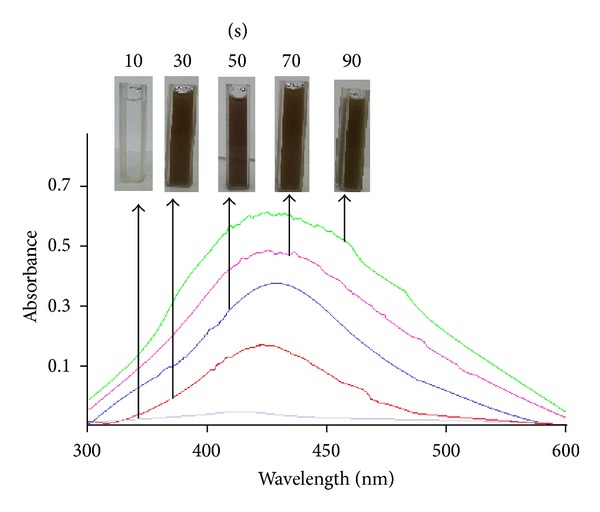
UV-visible spectrum showing the microwave irradiated silver nanoparticles synthesis recorded as a function of time.

For the synthesis of silver nanoparticles, 1 mL of pigment was added to 20 mL of AgNO_3_ and microwave-irradiated for a period of 90 s. The solution turned from colorless to brown within 90 s, and the absorption maximum between 400 and 450 nm increased steadily as a function of reaction time, caused due to surface plasmon resonance of silver nanoparticles in the visible region [17], which clearly indicates the preliminary synthesis of silver nanoparticles (Figure 1). 

**Figure 2:**
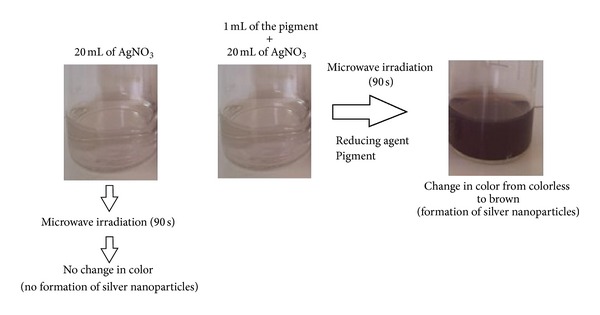
Pathway for the synthesis of silver nanoparticles.

For comparison purpose, 20 mL of AgNO_3_ solution was exposed to microwave irradiation, used as a blank. No change in color in this AgNO_3_ solution was observed, indicating that the pigment was the possible reducing agent (Figure 2). 

**Figure 3:**
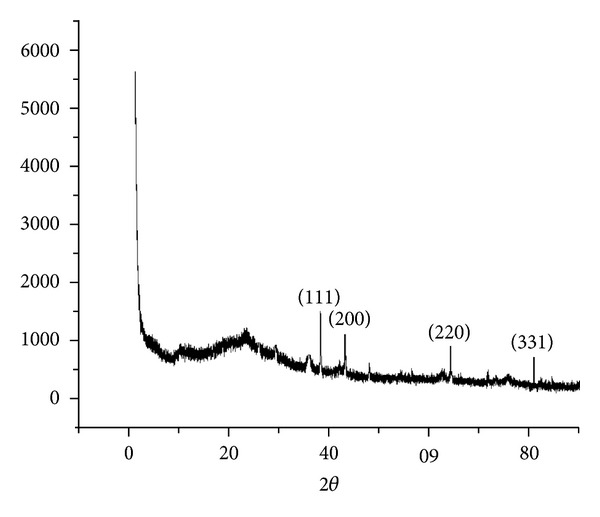
XRD pattern of synthesized silver nanoparticles.

**Figure 4:**
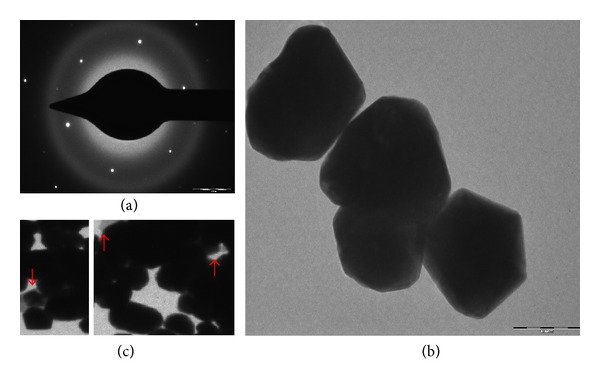
(a) Energy dispersive pattern image of silver nanoparticles. (b) TEM image of synthesized silver nanoparticles. (c) Nanoparticle shown with red arrow mark indicates the presence of organic moieties responsible for the stabilization and interbinding of silver nanoparticles.

The synthesis was further confirmed by X-ray diffraction.  Figure 3 shows a representative pattern of the synthesized nanoparticles after the reduction of AgNO_3_. Intense peaks corresponding to (111), (200), (220), and (311) were observed. These peaks can be indexed based on the FCC structure of silver (JCPDS files no. 03-0921), confirming the crystalline nature of the silver nanoparticles. Further the energy dispersive (ED) pattern of silver nanoparticle in Figure 4(a) reconfirms the crystalline nature of silver nanoparticles.

A representative TEM image is shown in Figure 4(b) which clearly indicates that silver nanoparticles were irregular in shape having an average size of 50 nm. The particles in this range are well known for having excellent antimicrobial activity [10]. On careful observation we can see a thin layer on the surface of nanoparticles (Figure 4(c)) (nanoparticles shown with red arrow mark) which indicates that these are the organic moieties that may be responsible for the stabilization and interbinding of silver nanoparticles. 

**Figure 5:**
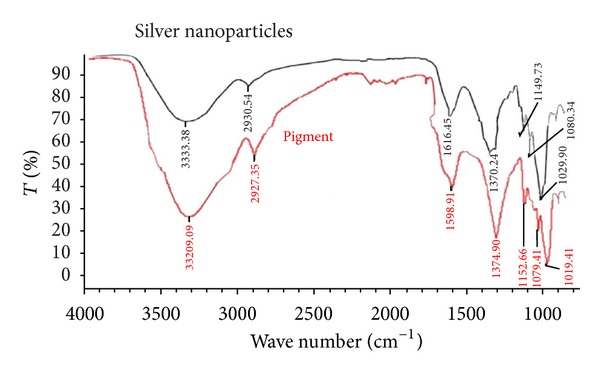
FTIR spectra of synthesized silver nanoparticles and pigment.

Figure 5 shows the FTIR spectra of the purified silver nanoparticles and pigment. The purified nanoparticles exhibited absorption peaks at 1149, 1616, 1645, and 3333 cm^−1^ due to cyclic C–O–C, C=O and OH functional groups, respectively. The peaks obtained were compared with pigment, and less intense peaks with slight shift were observed in the purified silver nanoparticles. From the FTIR spectra it may be inferred that the pigment was the probable reducing agent which was involved in the synthesis of silver nanoparticles. Further the pigment might have formed a layer on the silver nanoparticles (i.e., biological capping) that prevented the agglomeration of the particles. Thus, the nanoparticles were stabilized.

The exact mechanism of why pigment acts as reducing agent is still not clear; to our knowledge it may be the soluble nature of the pigment in water which makes it one of the best solvents for microwave heating. The pigment acts as reductant/stabilizer in the presence of microwave irradiation to assist the conventional solution-phase synthetic strategies based on redox reactions to prepare silver nanoparticles [18]. 

## 4. Conclusion 

In this study, we report rapid synthesis of silver nanoparticles in just 90 s by microwave irradiation method using pigment as a reducing agent. The XRD analysis showed the crystalline nature of silver nanoparticles, and the TEM image showed that the particles are irregular in shape having an average size of 50 nm which could be used as antimicrobial agent. FTIR studies show that the pigment is the reducing agent. The synthesis developed in this study has distinct advantages over biological and chemical methods in being rapid, safe, and nontoxic, and moreover the synthesis of silver nanoparticles by this route is the appropriate way to develop green technology for the bulk synthesis of silver nanoparticles for industrial purpose.
